# Commodity Recommendation Model Integrating User Psychological Data Analysis

**DOI:** 10.3389/fpsyg.2022.907865

**Published:** 2022-05-27

**Authors:** Yan Xu, Wantian Cui

**Affiliations:** Department of Economics, Liaoning University, Liaoning, China

**Keywords:** commodity recommendation, psychological data analysis, college students, integrating user, e-commerce recommendation

## Abstract

E-commerce recommendation plays an irreplaceable role in alleviating product information overload and improving consumers’ personalized experience and sales conversion rate. According to the idea of recommendation, e-commerce recommendation can be divided into two types: recommendation based on correlation and recommendation based on causality. The former, such as collaborative filtering and other recommendation methods, is highly dependent on data; the latter, such as product recommendation based on consumer psychology, has great superiority in new product recommendation due to the introduction of such domain knowledge as consumer psychology in the recommendation. In this paper, we select three representative consumer psychologies of “consumer motivation,” “consumer attitude,” and “consumer interest” to explore the recommendation of products with multiple consumer psychologies. On the one hand, according to the theory of consumption psychology and the need of e-commerce recommendation, some product-store attributes are selected as attribute variables. A comprehensive comparison and analysis of the patterns presented by multiple consumer psychology in product recommendations are conducted, and the patterns of multiple consumer psychology product recommendations are analyzed from two perspectives: recommendation stability and recommendation result patterns, respectively, and the reasons for them are analyzed. It is clear that the recommendation method based on dual consumption psychology and triple consumption psychology can also effectively achieve product recommendation. In this paper, we compare and analyze the difference in recommendation accuracy between single consumption psychology, dual consumption psychology, and triple consumption psychology and find that compared with single consumption psychology, product recommendation based on dual consumption psychology and triple consumption psychology can basically improve the accuracy of product recommendation, and the accuracy of dual consumption psychology is generally higher than that of triple consumption psychology, among which the accuracy of product recommendation based on dual consumption motivation–attitude psychology is the highest.

## Introduction

Consumer psychology believes that consumer psychology is the inner expression of consumer behavior, largely reflected through consumer behavior, consumer behavior is mostly governed by consumer psychology, and consumer psychology reflects the changes in the consumer’s mental activities throughout the consumption process, reflecting the consumer subject’s willingness to consume the consumer object, so the user’s consumer psychology can be obtained in certain ways, using the user’s consumer psychology to determine the user’s possible purchase behavior, and then achieve product recommendation (Strickland et al., 2020). At present, the research on consumption behavior and network consumption psychology is more in-depth and mature, and the network consumption psychology is mostly focused on “network consumption motivation, consumption attitude, purchase demand, interest, and purchase intention” (Fang et al., 2020). Most of them take network consumption psychology as the theoretical basis to analyze users’ personalized needs and realize personalized product recommendation for users. Based on the theoretical basis of consumer personality, it proves that consumer personality can realize high-precision product recommendation. In turn, the recommendation method and process are systematically analyzed from the perspective of consumer motivation, and it is concluded that consumer motivation psychology can realize high-precision product recommendation (Qian et al., 2019). With the rapid development of modern technology, the application of intelligent recommendation technology and user profiling system in the field of e-commerce is becoming more and more extensive (Wang, 2018; Jiang et al., 2019; Athique, 2020; Blair et al., 2021). Because intelligent recommendation technology not only brings users a good shopping experience, but also improves the efficiency of users’ shopping and greatly enhances product sales, which makes intelligent recommendation technology become a hot research topic in recent years (Cortez et al., 2018).

With the rapid development of the Internet economy, a large number of products have flooded into the e-commerce market, so that the intelligent recommendation has once again pushed to the wind (Cloarec et al., 2022). Although the development of the e-commerce industry is in full swing, even so, the development of many traditional e-commerce platforms is not ideal, these traditional e-commerce platforms cannot guarantee to provide comprehensive user analysis, and mining user needs to provide the ideal recommendation program, and the lack of close contact between the user and the platform (Cahyono et al., 2018; Cheng et al., 2019; Huang and Rust, 2021). In the background of “Internet+,” this paper designs and implements a product recommendation system by data mining the user data of the mall and using the technologies of user portrait and intelligent recommendation (Buyinza et al., 2020). The system not only can dig deeper into the user’s shopping needs, but also can establish a user portrait for the user according to the existing user data, realizing the true sense of a thousand people, and at the same time, on the basis of the user portrait combined with the recommendation technology to achieve the very ideal recommendation effect (Sabouhi et al., 2019). If e-commerce platforms want to attract new customers and retain old customers, they must understand the current needs of customers and dig deep into their potential needs and then recommend their products to customers according to their needs (James et al., 2019). With the hot development of Internet technology, recommendation system has become an indispensable part of almost every e-commerce platform and is even widely used in other fields, such as entertainment, food, film and television, culture, and education (Windels et al., 2020). When the scale of the enterprise reaches a certain level, there must be a mature recommendation system behind it for support. The recommendation system can recommend and display information that users may be interested in for a large amount of user data to improve the service quality of the system (Zahid et al., 2019). The product recommendation system is very powerful, and these platforms build user portraits for users through massive user data, analyze, and label users’ consumption behavior and habits, and at the same time with intelligent recommendation algorithms to select the most suitable product information from a large number of products to present to users (Kumar et al., 2018; Langlois and Elmer, 2019; Di Vaio et al., 2020; Shafqat et al., 2020). The emergence of user portrait technology is, in a sense, a great subversion of the traditional e-commerce industry. Therefore, it is very necessary to develop personalized recommendations about products based on users’ needs (Alic et al., 2019).

This paper describes the design and implementation of a product recommendation system based on user behavior and data. In order to improve the order rate of users for products and improve the problem of high redundancy of products on the platform and the complexity of users’ screening of products, the functions of the recommendation system are finely classified and analyzed. Based on the user data of the mall, this paper conducts an in-depth study of the user group and introduces the user portrait, intelligent recommendation, and other related technologies in detail based on reading and studying a large amount of literature. We also compare the recommendation effect of many common recommendation algorithms and propose a strategy based on the combination of user portrait and user preference to achieve accurate product recommendation based on the above research. The product recommendation system is a comprehensive project with a wide range of technical aspects, including user portrait technology, intelligent recommendation technology, web crawler technology, and data mining technology. Based on the above considerations, this paper intends to explore the law of multiple consumer psychology applied to product recommendation at the same time from the perspective of consumer psychology, that is, the law of product recommendation of multiple consumer psychology. In this paper, we choose the dual consumer motivation–attitude psychology, dual consumer motivation–interest psychology, and dual consumer attitude–interest psychology as the research objects and analyze the influence of dual consumer psychology (i.e., two kinds of consumer psychology applied to product recommendation process at the same time) on product recommendation; we take the triple consumer motivation–attitude–interest psychology as the triple consumer psychology research objects and analyze the triple consumer psychology (i.e., three kinds of consumer psychology). We also analyze whether the triple consumer psychology (i.e., three kinds of consumer psychology applied to the product recommendation process at the same time) can improve the accuracy of product recommendation. In this paper, we select three representative consumer psychologies of “consumer motivation,” “consumer attitude,” and “consumer interest” to explore the recommendation of products with multiple consumer psychologies. On the one hand, according to the theory of consumption psychology and the need of e-commerce recommendation, some product-store attributes are selected as attribute variables, and the information of purchased products is discretized.

## Materials and Methods

### Participants

Real-time computing differs from offline computing in that the results of real-time computing reflect more of the current user’s preferences for products in the recent past than offline computing. The offline recommendation result is the result of analyzing all the past behavioral data of users. At different points in time, users show changes in their preferences for products. For example, if user a gives a very high rating to product p at a certain point in time, then at that point in time, the user is interested in other products that are very similar to product p. Conversely, at the same point in time, the user is interested in other products that are very similar to product p. Conversely, if at the same point in time, user a gives a very low rating to product k, then in the nearest time, user a will probably not be interested in products similar to product k either. Therefore, what we need to do in real-time recommendation is that after a user rates a product, the recommendation result will be updated according to the user’s recent rating data, so that the recommendation result of the product will be closer to the current user’s preferences and meet the user’s recommendation needs. The user’s recent score is only in offline recommendation. The ALS algorithm uses the user’s score table, and the user’s recent score only updates a small part of the score table. Therefore, the recommendation result using the ALS algorithm does not change significantly. Users do not feel the obvious changes of recommended products, which greatly affects the user experience. In addition, real-time recommendation requires more strict time efficiency, so that too many iterations cannot occur to reduce the time needed for calculation and improve user experience. So it will reduce the requirement of recommendation accuracy. In general, the recommended product results will change significantly after the user’s rating, or after the last few ratings, and the real-time calculation should not take too long to run, and the amount of calculation should be reduced.

### Design

When a user rates a product, the current recommendation result is changed once for that user, and the system selects the k most similar products as candidates. The “recommendation priority” of the candidate products is the main factor that influences the priority of the recommendation to the user. Based on the user’s last ratings, the recommendation priority of these products is calculated for the user. After that, the newly obtained data results are updated with the last recommended data results in the database according to the recommended priority, and the combined product recommendations are selected as the latest data. Specifically, the rating table in the database is used to obtain the latest rating data *K* of this user for the product, and these products are put into the set *RK*, while the k products with the highest similarity to the product P are then obtained from the product similarity table and noted as *S*. Each item in *S* is denoted as *q*; each item in *RK* is denoted as *r*; the recommendation priority of each item in the set S is calculated by the formula *E*.


(1) $$E=\log\left(incount,1\right)-\log\left(recount,1\right)+\frac{\sum sim\left(q,r\right)}{sum\_R_{r}}$$

where: *Rr* denotes the rating of item r by user u.

(*q, r*) denotes the similarity between alternative goods q and goods r. The minimum similarity between goods is set to 0.6, and when the similarity between two goods is lower than 0.6, it is judged that there is no similarity between the two goods and is ignored.

*sim_sum* denotes the number of alternative goods q with similarity greater than 0.6 to good r in *RK*.

incount indicates the number of items in the RK that match the similarity to the alternative item q by more than 0.6 and whose own rating is not less than 3.

In the first step, when the similarity is higher than a predetermined value, the rating of the item is multiplied by the similarity between the two items, the sum of all the products is divided by the number of items with similarity higher than the predetermined value, and the result is used as the predicted rating of the alternative item by the current user.


(2) $$E_{r}=\frac{\sum sim\left(q,r\right)}{sum\_R_{r}}\times R_{r}$$

Then, the number of items in the user’s product r with similarity greater than 0.6 to the alternative item q and whose own rating is not less than 3 is denoted as *incount*, and the calculation of lg *maxincount, 1* to increase the weight of users’ highly rated goods in the calculation of the product recommendation priority, the fundamental purpose is to increase the recommendation priority for good q when the alternative good q is similar to the goods with high recent rating data of the current user and has a higher chance of being liked by the current user, and then, the recommendation priority for good q increases by lg *maxincount, 1*. If the alternative item q is more similar to the current user’s most recent K highly rated items of the item, which is also an indication that q has a high probability of receiving high ratings, then item q should be recommended more, so the recommendation priority is enhanced by a larger amount; conversely, when the alternative item q is less similar to the current user’s most recent K highly rated items, the recommendation priority of the alternative item will not be greater. Then, the number of items in user’s product r with similarity greater than 0.6 to alternative item q and own rating less than 3 is noted as *recount*, and lg *max* is calculated *recount, 1* to reduce the weight of the user’s review score items in the calculation of the product recommendation priority, and the fundamental purpose is that when the alternative item q is similar to the item with very low recent user rating data similar to the one with a higher chance of being resented by the current user, then the recommendation priority for item q is reduced by lg *maxrecount, 1*.

If the alternative product q is more similar to the current user’s most recent K rated products with low ratings, it also means that product q has a high probability of getting low ratings, so product q should not be recommended, so the recommendation priority is weakened more; on the contrary, when the alternative product q is less similar to the current user’s most recent K rated products, the recommendation priority of the alternative product will not be the alternative product’s recommendation priority which is not reduced significantly.

### Measures

The premise of the real-time recommendation algorithm is as follows:

(1) The real-time algorithm can quickly obtain the rating data of all users for a product by intercepting the latest K ratings in the database and sending it to the calculation module.
(2) Before the real-time recommendation, the similarity matrix of the products has been calculated by the ALS algorithm in the offline recommendation.
(3) Flume gets the user behavior data through buried points and pushes the real-time scoring data to the Kafka message queue.

The algorithm process is as follows: After users rate the products, the calculation module extracts the users’ rating data and the product data with high product similarity in the database, transmits them according to the fixed information format, and gets the similarity data of related products through the preset calculation module, which is pushed to the product recommendation page to update the product categories in real time according to the product priority.

After the front-end page gets the user’s rating behavior, the rating data will be inserted into the non-relational database in a set format, and the corresponding data content will be obtained by extracting the data recorded in the rating table when the corresponding calculation is performed.

In the offline algorithm, the ALS algorithm has already obtained the similarity matrix between products, so it is easy to obtain K alternative similar products with the real-time rating product p: read the product data in the mall from the database, and in order to get the set of products with the most similar product id, we need to obtain the corresponding data from simhash and output the corresponding products according to the similarity between products. The set of products is output according to the similarity between products. These products are arranged in positive order according to the similarity, the higher the similarity in the first rank, and the obtained product collection is renamed candidate products to build a table and wait for the subsequent data push.

After the latest product recommendation priority list is calculated, these data are transferred to the back-end database, the user’s previous product recommendation list is read from the database, the two tables are merged to produce a new product recommendation table, and the product with the highest recommendation is re-selected to update the product results on the product recommendation page.

## Multiple User Psychological Data Analysis

### Personalized Attribute Information Acquisition

The acquisition of commodity-store attributes is the basis for conducting personalized recommendation research. In this paper, the acquisition of attributes is based on user consumption history list records, combined with attribute discretization standard rules to achieve discrete value acquisition for general commodities, but in the actual process, because commodity recommendations based on consumer psychology are recommended on an individual basis, the user’s own feelings and preferences are the most important reference requirement and test in the process. For example, the definition of beauty *X*6 needs to be more accurate discrete values with the help of communication with users, given its individual differences. Therefore, in this paper, the data collection rules are adjusted individually for personalized information with the help of user interviews in order to further improve the rationality of discrete processing of product attributes.

Table 1 shows the user interview questions for the example of apparel products. The questionnaire survey was conducted for the discrete values of product attributes that may hide the subjective perception of users. If the match between the discrete values of the preset attributes and the interview results is higher than 70%, it means that the attribute setting of the product is in line with the user’s cognition; if the comparison result is lower than 70%, it means that the user has personalized cognition of the product attributes and needs to adjust the attribute setting rules according to the user’s thinking until the comparison result. The comparison result is higher than 70%. The purpose of this interview is to test whether the discrete processing of the product attributes is in line with the user’s cognition and to make personalized adjustments in response to the test results, highlighting the experimental guideline of focusing on the user’s needs. Through this process, the product-store attribute matrix can be more accurately established, providing a guarantee for the accuracy and authenticity of subsequent personalized recommendations. It also facilitates user interviews on the reasons for users’ purchases to further determine the rationality of consumer psychology while user interviews on the comparison criteria of the product recommendation results to test their recommendation accuracy.

**TABLE 1:** List of user interview questions.

Nature of the problem	Specific questions	Rating percentage (SD)	Option settings		
General	Online shopping habits	25	Excellent	Good	General
	Online Shopping Reviews	25			
	Online shopping quality	25			
	Online shopping packaging	25			
Targeted Questions	Subjective evaluation	10	Excellent	Good	General
	Objective evaluation	20			
	Price	20			
	Services	20			
	After Sales Service	10			
	Personalization	20			

*SD: standard deviation.*

According to the research process of product recommendation based on consumer psychology, consumer psychology needs to satisfy the conditions of “can be reflected by the products purchased,” “can be analyzed by Bayesian network method,” and “can realize product recommendation.” Therefore, this paper proposes four criteria to determine whether consumer psychology is applicable to the research of product recommendation based on consumer psychology.

Whether consumer psychology can be expressed through goods. It refers to whether a certain psychological characteristic of the user can be expressed through the commodity, such as through the essential attributes of the commodity, other attributes of the commodity, etc. It is the expression of the consumer psychology positioning a single commodity. The user’s consumption motivation can be positioned through the price and brand of the goods purchased by the user and has the expressiveness of the goods; the consumer attitude is expressed by the concept of goods embodied in the purchase process and can be positioned psychologically through the attributes of the goods, consumer behavior, etc.; the consumer interest is directly expressed in the user’s preferred needs in the consumption target and can be positioned by the type of goods consumed. Consumer interest is difficult to locate in each product because it is reflected in the consumer behavior and characteristics in the specific consumption process, which is difficult to be reflected in the consumption results. Consumer attitudes in consumer personality can be portrayed from the commodity perspective, but user purchasing style is a study of user browsing records and specific behaviors in the purchasing process, which is more focused on the user perspective rather than the commodity perspective. Consumption ability can be reflected by the quality of the purchased goods, the degree of familiarity with the goods, and other information about their commodity expressiveness.

Finally, it is also necessary to consider the accuracy of the recommendation. That is, whether the product recommendation can be accurately achieved, which mainly examines whether the consumer psychology itself can effectively distinguish different goods, whether it can effectively capture the common features of consumers’ purchase of goods, etc., mainly reflected in the detailed and specific degree of consumer psychology theory, the consumer psychology with more classifications, the product recommendability will generally also be very high. Therefore, these seven consumer psychologies in the consumer consumption process, obviously embody the recommendability of each different, mainly can be summarized as shown in Table 2.

**TABLE 2:** Recommended attributes for different consumer psychology.

Consumer psychology	Nature of product	Product properties	Recommended model matching	Recommended accuracy (SD)
Motivation theory	181	3.69	0.58	2.69
Interest Theory	196	3.25	0.36	
Attitude Theory	69	3.25	0.26	
Purchase Method	95	3.69	0.29	
Temperament Theory	135	3.26	0.59	2.51
Capability Theory	159	3.21	0.67	
Demand Theory	96	3.49	0.67	

*SD: standard deviation.*

In this paper, we choose to explore the influence of consumer psychology on product recommendation from the perspective of three kinds of consumer psychology: consumer motivation, consumer attitude, and consumer interest. The consumer motivation *C*1 is divided into cheap motivation, famous motivation, new motivation, same motivation, realistic motivation, and convenient motivation, while the consumer attitude *C*2 is divided into frugal and practical, novel and free, conservative and cautious, eccentric and paranoid, and compliant and accepting from the user’s attitude toward the shopping process, and the consumer interest *C*3 is divided into brand, quality, comprehensive, and aesthetic from the consumer interest perspective, as shown in Table 3.

**TABLE 3:** Information on consumer psychological attributes.

Variable Name	Variable values					
Consumer Motivation	Motivation to seek integrity	Motivation for seeking a name	Motivation to seek new	Motivation to seek common ground	Motivation to seek truth	Motivation
Consumer Attitude	Frugal and practical	Novelty and freedom	Conservative and cautious	Quirky Paranoia	Compliant Acceptance	/
Consumer Interest	Brand Type	Quality type	Comprehensive	Aesthetic type	Value-based	Price Type

### Product Recommendation Model

First, 50 randomly selected products are discretized by product attributes, and the data matrix is input into the Bayesian network structure to apply the Bayesian network inference method for product recommendation and judge whether each Bayesian network structure meets the user’s personalized consumption psychology. The product-store attributes are discretized and processed to obtain the attribute matrix, and the posterior probability of each product is output through BNT toolbox, taking eight products as an example. The main recommended products under multiple consumption psychology are the common set of product recommendations under three single consumption psychology, which meet all the recommendation conditions of consumption psychology before they become the recommended candidates under triple consumption psychology.

**FIGURE 1:**
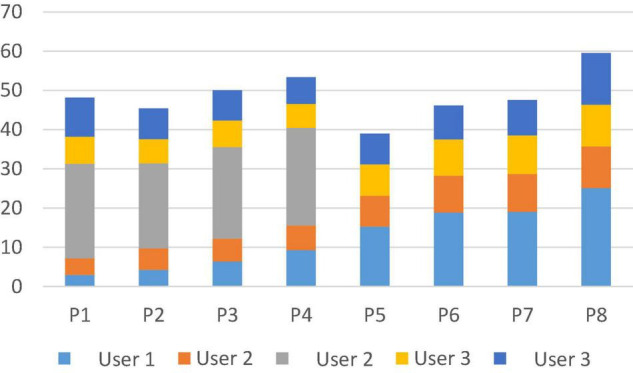
Triple consumer psychology check rate comparison line chart.

From the overall user’s triple consumer psychology detection rate, compared with the single consumer psychology detection rate, it can also be seen in Figure 1 that the detection rate of triple consumer psychology recommendations is higher than that of single consumer psychology in most cases, indicating that the combination of triple consumer psychology gets a higher detection rate than single consumer psychology recommendations. However, compared with the dual consumption psychology, the advantage of triple consumption psychology is not obvious. From the comparison of the mean value of the detection rate, it is found that the detection rate of triple consumption psychology is lower than the detection rate of dual recommendation of consumption motivation and consumption attitude *P*12 and lower than the detection rate of dual recommendation of consumption motivation and consumption interest *P*13, and the overall detection rate still has a decreasing trend. Consumer psychology-based product recommendation uses the more stable personality psychological characteristics of users and focuses on the user’s shopping psychological experience and target expectations from the user’s perspective, which is a more important recommendation method in e-commerce recommendation systems. Consumer attitudes in consumer personality can be portrayed from the commodity perspective.

From the abovementioned triple consumption psychology recommendation process and results, it is found that the number of recommended products is significantly reduced, which indicates that this method will improve the accuracy of recommendation results by reducing the number of products that users are not sure to buy; at the same time, this method only retains the products that users are willing to buy under the three consumption psychologies and screens out other products, which will also reduce the rate of product recommendation checking and may miss the products that users are willing to buy.

**FIGURE 2:**
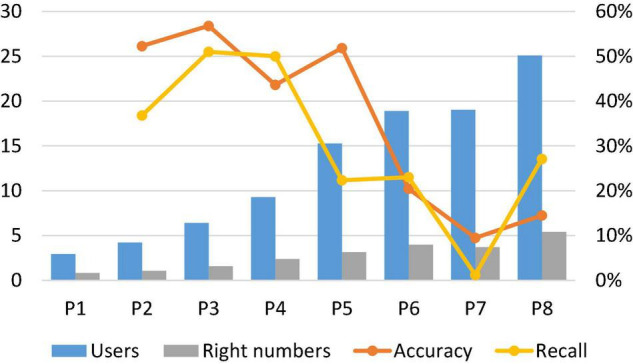
Mean and variance of the triple motivation–attitude–interest psychological recommendation check.

Figure 2 shows the mean and variance of the results of the triple consumption psychology recommendation check rate using multi-user data, mainly comparing and analyzing the overall situation of the check rate of different consumption psychologies. Among them, *P*123 represents the recommendation accuracy rate under the triple psychology of consumption motivation, consumption attitude, and consumption interest. From the viewpoint of the mean of the detection rate, the recommended detection rate of *P*123 under the triple consumption psychology of consumption motivation, consumption attitude, and consumption interest is higher than the detection rate of the three types of single consumption psychology, which basically achieves the improvement of the detection rate. That is, whether the product recommendation can be accurately achieved, which mainly examines whether the consumer psychology itself can effectively distinguish different goods, whether it can effectively capture the common features of consumers’ purchase of goods, etc.

### Stability Analysis of Recommendation Results Under Multiple Consumer Psychology

**FIGURE 3:**
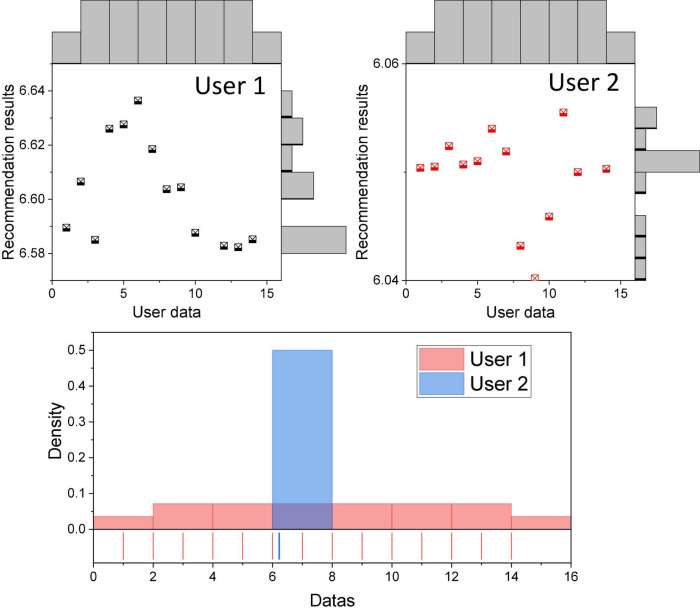
Differences in recommendation results by user.

Consumer psychology-based product recommendation uses the more stable personality psychological characteristics of users and focuses on the user’s shopping psychological experience and target expectations from the user’s perspective, which is a more important recommendation method in e-commerce recommendation systems. In the process of multiple consumer psychology product recommendation, it is found that the stability of consumer psychology-based e-commerce recommendation is high from the recommendation process, and the recommendation stability is mainly reflected in the association of the same user attribute node and category node, that is, the stability of Bayesian network structure, and also in the accuracy of the product recommendation results of different users with small fluctuations. To further demonstrate such variability, three different users were randomly selected, and their multiple product recommendation results were collected and averaged. From the descriptive indexes of the three groups of sample data in Figure 3, the means and standard deviations are different, but the differences are small, the possibility of having significant differences is small, and it is judged that there are no significant differences among the three groups of samples.

Stability is also reflected in the existence of stability of probabilistic combinations of consumer personality psychological traits learned based on Bayesian networks. The stability of the same user’s consumption psychology is proved longitudinally. Since different consumers have different consumption psychology, the validation of the stability of consumption psychology continues to randomly select n users, obtain the type of consumption psychology of the user under different base data by cross-validation of the user’s data, and compare and analyze to verify whether the user’s consumption psychology is stable. In order to make full use of the data, the 4-fold cross-validation method is chosen to validate the triple consumption psychology recommendation method.

## The Stability of Recommendation Results Corresponds to the User’s Psychological Data

For the recommendation stability law presented in the process of multiple consumer psychology recommendation, the fundamental reason is that the actual consumer psychology type of the user has the characteristic of stability, the user consumption process to buy a wide range of commodity categories, but based on the commodity attribute information to learn the user’s consumer psychology type is stable, the user’s consumer psychology in a certain period of time changes less, which is conducive to the stability of consumer psychology with the help of the analysis. This is conducive to analyzing their purchase behavior with the stability of consumer psychology, judging the goods that users are willing to buy, and making e-commerce recommendations for them. The law also reflects that consumer psychology dominates users’ consumption behavior. We can learn the type of users’ consumption psychology from their purchased goods records and learn the deep-seated psychological reasons for their purchases, so that we can better analyze the common attributes of their purchases through different goods and better recommend them to users. The ALS algorithm used in offline recommendation takes longer time in iterative operation, which is not suitable for real-time recommendation, and in offline recommendation, the ALS algorithm uses the user’s rating table of the product.

**FIGURE 4:**
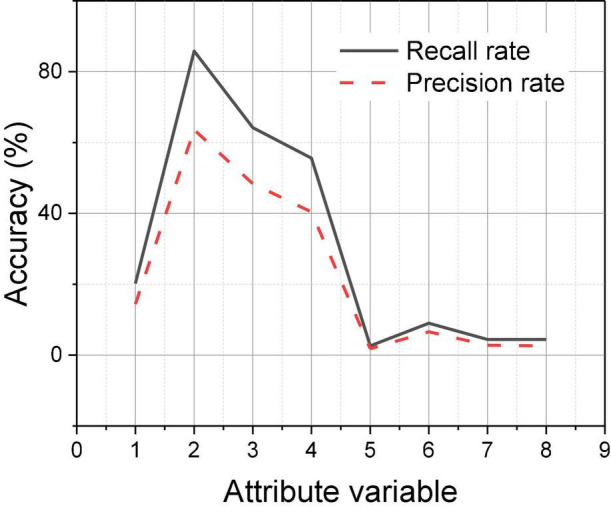
Predicted trends in the results of multiple consumer psychological recommendations.

From the perspective of the variance of the check-all rate, the overall fluctuation is relatively small, in which the overall fluctuation of the triple consumption psychology check-all rate is the largest, the overall fluctuation of the single consumption psychology is smaller, and the law can be summarized as the single consumption psychology recommendation check-all rate value fluctuation is smaller than the overall fluctuation of the double consumption psychology check-all rate. In summary, it can be predicted that the check rate of multiple consumption psychology decreases with the increase in consumption psychology type, as shown in Figure 4, showing a decreasing trend; the recommended check rate of goods of multiple consumption psychology reaches a peak when double consumption psychology and then decreases with the increase in consumption psychology. If real-time recommendation continues to use ALS algorithm in offline recommendation, it does not have the ability to get new recommendation results in real time because of the huge running time of the algorithm.

**FIGURE 5:**
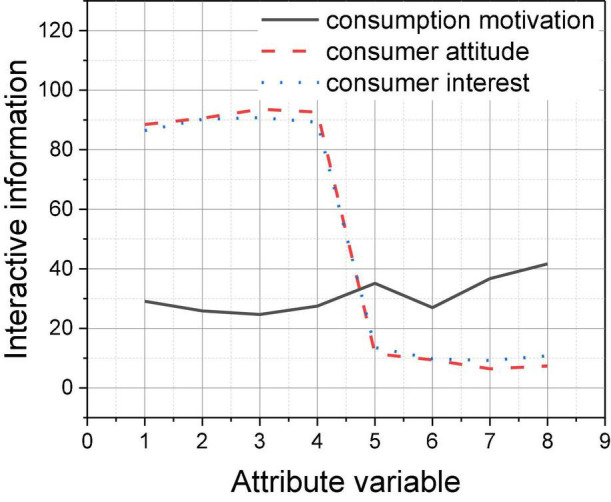
Mutual information value of each consumer psychology and attribute node.

The comparison analysis reveals that among the three types of consumer psychology, consumer motivation theory involves the largest number of product-attribute variables, so its content is the most accurate, so the accuracy of consumer motivation recommendation check is the highest, followed by consumer attitude, and the accuracy of consumer interest recommendation is the lowest in comparison. From the perspective of dependency, that is, the size of mutual information, the mutual information value of consumer motivation psychology and commodity-store attribute nodes is stronger in general, as shown in Figure 5, 1–14 represent fourteen commodity-store attribute nodes, and the values in the table are the mutual information values of each consumer psychology and attribute nodes; it can be found that consumer motivation psychology is basically located above other consumer psychology, indicating that consumer motivation and commodity-store attribute nodes. If the dependency relationship is stronger, the possibility of achieving accurate e-commerce recommendation to users through this perspective is stronger. By comparing the two prediction methods, Bayesian network method requires the type of consumer psychology to be able to interpret the content from the perspective of commodity attributes, all kinds of different consumer psychology can achieve is the effective differentiation of commodity-store attributes, and experimental group increased significantly more than those of the control group.

From the full text, we can see that the basis of obtaining the type of consumer psychology in this paper is user purchase records. When more consumer psychology is known, the information in the data is basically analyzed, and over-interpretation will lead to the trap of “over-fitting,” which will interfere with the whole product recommendation process and is not conducive to determining the real preference of users, so it may be better to learn two consumer psychologies from the same data to improve the recommendation accuracy. So some consumer psychology such as consumption ability is difficult to achieve the Bayesian network-based commodity recommendation method, and also, there will be different consumer psychology recommendability which will be different.

The product recommendation under multiple consumer psychology will also have the effect of combining consumer psychology with recommendation to further enhance recommendability, mainly because the greater the difference in theoretical perspectives among consumer psychology and the greater the difference in consumer psychology content, the more likely it is to obtain comprehensive consumer psychology information of users and better recommend to users. The above pattern is mainly related to the exhaustive degree of consumer psychology theory, the strength of the relationship between consumer psychology and product attributes, and data mining. The more detailed the content of consumer psychology, the greater the value of mutual information with commodity attributes, the more likely it is to have higher recommendability, so the dual consumer attitude–motivation psychology has the best recommendability. However, the useful information in the data, once fully mined, will not increase substantially with more data mining and analysis, so triple consumption psychology product recommendation is not better than double consumption psychology.

## Conclusion

This paper applies the theory of consumer psychology to e-commerce recommendation, uses the Bayesian network method as a tool, investigates the product recommendation law based on dual consumer psychology and the product recommendation law based on triple consumer psychology, and deeply explores the influence of the combination of multiple consumer psychologies on product recommendation to provide reference for the research in the field of consumer psychology recommendation. The information of product-store attributes is discretized, and the association between product-store attributes and consumer psychology is established, laying the foundation for building a Bayesian network with consumer psychology as category nodes and product-store attributes as attribute nodes, and also analyzing the combination method of multiple consumer psychologies in the Bayesian network. We explore the product recommendation law of dual consumption psychology, summarize the law of combining dual consumption psychology on recommendation results, focus on analyzing the changes in recommendation check-all rate and check-accuracy rate, and prove the feasibility of the product recommendation method based on dual consumption psychology. Research on the laws of triple consumption psychology in product recommendation shows that when more consumer psychology is known, the information in the data is basically analyzed, and over-interpretation will lead to the trap of “over-fitting,” which will interfere with the whole product recommendation process and is not conducive to determining the real preference of users. In this thesis, when there are duplicate product ids in the last product recommendation priority table and the current product recommendation priority, the recommendation priority of these products is selected and updated to the latest product recommendation priority. Finally, the top K products are selected as the result according to the recommendation priority in the product recommendation table, and the product page seen by the user is updated to get the final recommendation result. The final recommendation result is obtained.

## Data Availability Statement

The original contributions presented in the study are included in the article/supplementary material, further inquiries can be directed to the corresponding author.

## Ethics Statement

Ethical review and approval was not required for the study on human participants in accordance with the local legislation and institutional requirements. Written informed consent from the patients/participants was not required to participate in this study in accordance with the national legislation and the institutional requirements.

## Author Contributions

YX and WC have made a direct and intellectual contribution to this manuscript. Both authors contributed to the article and approved the submitted version.

## Conflict of Interest

The authors declare that the research was conducted in the absence of any commercial or financial relationships that could be construed as a potential conflict of interest.

## Publisher’s Note

All claims expressed in this article are solely those of the authors and do not necessarily represent those of their affiliated organizations, or those of the publisher, the editors and the reviewers. Any product that may be evaluated in this article, or claim that may be made by its manufacturer, is not guaranteed or endorsed by the publisher.

## References

[B1] Alic A. S. Almeida J. Aloisio G. Andrade N. Antunes N. Ardagna D. (2019). BIGSEA: a big data analytics platform for public transportation information. *Future Gener. Comput. Syst.* 96 243–269. 10.1016/j.future.2019.02.011

[B2] Athique A. (2020). Integrated commodities in the digital economy. *Media Cult. Soc.* 42 554–570. 10.1177/0163443719861815

[B3] Blair G. Christensen D. Rudkin A. (2021). Do commodity price shocks cause armed conflict? A meta-analysis of natural experiments. *Am. Political Sci. Rev.* 115 709–716. 10.1017/S0003055420000957

[B4] Buyinza J. Nuberg I. K. Muthuri C. W. Denton M. D. (2020). Psychological factors influencing farmers’ intention to adopt agroforestry: a structural equation modeling approach. *J. Sustain. For.* 39 854–865. 10.1080/10549811.2020.1738948

[B5] Cahyono A. E. Kurniawan M. U. Sukidin S. Kantun S. (2018). Community empowerment models of tourism village based on superior commodities: realizing economic resilience. *J. Distrib. Sci.* 16 29–36. 10.15722/jds.16.11.201811.29

[B6] Cheng X. Gu Y. Shen J. (2019). An integrated view of particularized trust in social commerce: an empirical investigation. *Int. J. Inf. Manag.* 45 1–12. 10.1016/j.ijinfomgt.2018.10.014

[B7] Cloarec J. Meyer-Waarden L. Munzel A. (2022). The personalization-privacy paradox at the nexus of social exchange and construal level theories. *Psychol. Mark.* 39 647–661. 10.1002/mar.21587

[B8] Cortez C. T. Saydam S. Coulton J. Sammut C. (2018). Alternative techniques for forecasting mineral commodity prices. *Int. J. Min. Sci. Technol.* 28 309–322. 10.1016/j.ijmst.2017.09.001

[B9] Di Vaio A. Palladino R. Hassan R. Escobar O. (2020). Artificial intelligence and business models in the sustainable development goals perspective: a systematic literature review. *J. Bus. Res.* 121 283–314. 10.1016/j.jbusres.2020.08.019

[B10] Fang Y. Wang H. Zhao L. Yu F. Wang C. (2020). Dynamic knowledge graph based fake-review detection. *Appl. Intell.* 50 4281–4295. 10.1007/s10489-020-01761-w

[B11] Huang M.-H. Rust R. T. (2021). A strategic framework for artificial intelligence in marketing. *J. Acad. Mark. Sci.* 49 30–50. 10.1007/s11747-020-00749-9

[B12] James P. B. Wardle J. Steel A. Adams J. (2019). Post-Ebola psychosocial experiences and coping mechanisms among Ebola survivors: a systematic review. *Trop. Med. Int. Health* 24 671–691. 10.1111/tmi.13226 30843627

[B13] Jiang C. Rashid R. M. Wang J. (2019). Investigating the role of social presence dimensions and information support on consumers’ trust and shopping intentions. *J. Retail. Consum. Serv.* 51 263–270. 10.1016/j.jretconser.2019.06.007

[B14] Kumar D. S. Purani K. Viswanathan S. A. (2018). Influences of ‘appscape’ on mobile app adoption and m-loyalty. *J. Retail. Consum. Serv.* 45 132–141. 10.1016/j.jretconser.2018.08.012

[B15] Langlois G. Elmer G. (2019). Impersonal subjectivation from platforms to infrastructures. *Media Cult. Soc.* 41 236–251. 10.1177/0163443718818374

[B16] Qian Y. Zhang Y. Ma X. Yu H. Peng L. (2019). EARS: emotion-aware recommender system based on hybrid information fusion. *Inf. Fusion* 46 141–146. 10.1016/j.inffus.2018.06.004

[B17] Sabouhi F. Bozorgi-Amiri A. Moshref-Javadi M. Heydari M. (2019). An integrated routing and scheduling model for evacuation and commodity distribution in large-scale disaster relief operations: a case study. *Ann. Oper. Res.* 283 643–677. 10.1007/s10479-018-2807-1

[B18] Shafqat S. Kishwer S. Rasool R. U. Qadir J. Amjad T. Ahmad H. F. (2020). Big data analytics enhanced healthcare systems: a review. *J. Supercomput.* 76 1754–1799. 10.1007/s11227-017-2222-4

[B19] Strickland J. C. Campbell E. M. Lile J. A. Stoops W. W. (2020). Utilizing the commodity purchase task to evaluate behavioral economic demand for illicit substances: a review and meta-analysis. *Addiction* 115 393–406. 10.1111/add.14792 31454109

[B20] Wang S.-T. (2018). Integrating KPSO and C5.0 to analyze the omnichannel solutions for optimizing telecommunication retail. *Decis. Support Syst.* 109 39–49. 10.1016/j.dss.2017.12.009

[B21] Windels K. Champlin S. Shelton S. Sterbenk Y. Poteet M. (2020). Selling feminism: how female empowerment campaigns employ postfeminist discourses. *J. Advert.* 49 18–33. 10.1080/00913367.2019.1681035

[B22] Zahid H. Mahmood T. Morshed A. Sellis T. (2019). Big data analytics in telecommunications: literature review and architecture recommendations. *IEEE CAA J. Autom. Sin.* 7 18–38. 10.1109/JAS.2019.1911795

